# Acute Effect of Transcutaneous Auricular Vagus Nerve Stimulation in Two Different Locations on Blood Pressure and Cardiac Autonomic Modulation in Healthy and Hypertensive Individuals: Pilot Study of a Randomized Crossover Clinical Trial

**DOI:** 10.1002/pri.70209

**Published:** 2026-04-07

**Authors:** Paulo Henrique Leite Souza, João Carlos Ferrari Corrêa, Bruno Paulino Venâncio, Liége Sanches Buzá, Gustavo Oliveira da Silva, Felipe Fregni, Fernanda Marciano Consolim‐Colombo, Raphael Mendes Ritti Dias, Fernanda Ishida Corrêa

**Affiliations:** ^1^ Doctoral and Master Program in Science of Rehabilitation Nove de Julho University São Paulo Brazil; ^2^ Neuromodulation Center and Center for Clinical Research Learning, Harvard Medical School Spaulding Rehabilitation Hospital and Massachusetts General Hospital Boston Massachusetts USA

**Keywords:** arterial pressure, autonomic nervous system, hypertension, transcutaneous auricular vagus nerve stimulation

## Abstract

**Background:**

and PurposeTranscutaneous auricular vagus nerve stimulation (taVNS) has emerged as a promising noninvasive strategy to modulate autonomic function and reduce blood pressure (BP). However, whether the stimulation site influences acute cardiovascular responses remains unclear.

**Objective:**

To compare the acute effects of taVNS applied to the tragus and cymba conchae on BP and cardiac autonomic modulation in hypertensive and healthy individuals.

**Methods:**

This pilot randomized crossover clinical trial included 14 hypertensive and 14 healthy participants who underwent two taVNS sessions (tragus and cymba conchae) in randomized order, one week apart. Sessions lasted 30 min and delivered a sinusoidal current (30 Hz, 500 ms pulse width), with intensity adjusted to the individual sensory threshold. Systolic BP (SBP), diastolic BP (DBP), and heart rate variability (HRV) parameters were assessed at baseline, during stimulation, and post‐stimulation. Generalized estimating equations were used for analysis.

**Results:**

A significant group × time × stimulation site interaction was observed for SBP (*χ* ^2^ = 19.0, *p* = 0.001) and DBP (*χ* ^2^ = 10.0, *p* = 0.038). Cymba conche stimulation reduced BP in healthy individuals, whereas no meaningful BP changes were observed in hypertensive participants. Tragus stimulation reduced diastolic BP only in the hypertensive group. Significant interactions were also detected for normalized spectral HRV components and sample entropy (*p* < 0.05), though autonomic responses were modest and not consistently indicative of enhanced parasympathetic modulation.

**Discussion:**

Acute taVNS induces site‐ and group‐dependent cardiovascular responses, with limited clinical impact after a single session. These findings suggest that short‐term stimulation may be insufficient to overcome impaired autonomic regulation in hypertension and highlight the need for repeated or longer stimulation protocols in future clinical trials.

## 1 Introduction

Hypertension is a highly prevalent chronic condition, affecting more than one billion people worldwide (Burnier and Egan 2019). Untreated hypertension has been associated with adverse clinical outcomes, including cardiovascular and renal complications, as well as reduced quality of life and life expectancy, representing a significant public health concern (Barroso et al. 2021; Brouwers et al. 2021; Péres et al. 2003). Despite the wide availability of pharmacological therapies, less than 20% of hypertensive individuals achieve adequate blood pressure (BP) control with low adherence to treatment and resistance to medication intake being determining factors for this therapeutic limitation (Coelho et al. 2024). In this context, adjunctive strategies alongside pharmacotherapy may prove beneficial (Carandina et al. 2021).

Transcutaneous auricular vagus nerve stimulation (taVNS) is a non‐invasive neuromodulation technique that involves applying electrical stimulation to specific regions of the external ear innervated by afferent fibers of the auricular branch of the vagus nerve, with the aim of modulating central autonomic circuits (Farmer et al. 2020; Yap et al. 2020). Preliminary evidence suggests that taVNS may be associated with beneficial effects on cardiovascular function, including acute reductions in sympathetic nerve activity (Clancy et al. 2014) and increases in cardiac baroreflex sensitivity in healthy individuals (Antonino et al. 2017), acute reductions in blood pressure in individuals with hypertension (da Silva et al. 2019), and improvements in autonomic balance and endothelial markers in individuals with metabolic syndromes (de Moraes et al. 2023).

Despite advances in research on the therapeutic effects of taVNS, there is a need for standardization of stimulation parameters, particularly concerning defining the most suitable anatomical region for electrode placement. Among the most studied areas, the tragus and the cymba conchae stand out, frequently used in experimental and clinical protocols. The tragus is a cartilaginous prominence situated anterior to the external auditory canal, containing approximately 45% of the vagus nerve projections (Badran et al. 2018; Peuker and Filler 2002). In contrast, the cymba conchae is described as predominantly innervated by the auricular branch of the vagus nerve (Peuker and Filler 2002). Notwithstanding variations in vagal innervation density, both regions have been utilized for taVNS, with evidence demonstrating that stimulation of either area can reduce blood pressure and enhance cardiac autonomic regulation (de Moraes et al. 2023; da Silva et al. 2019). However, studies that directly compared these stimulations in the same sample are unavailable.

The present study aimed to compare the acute effects of taVNS applied to the tragus and cymba conchae’s regions on BP and cardiac autonomic modulation in subjects with hypertension and healthy participants. The hypothesis is that taVNS in the cymba conchae exerts superior effects on blood pressure and cardiac autonomic modulation in hypertensive individuals due to the greater density of afferent fibers of the auricular branch of the vagus nerve in this region, which project directly to the nucleus of the solitary tract in the brainstem.

## 2 Methods

### 2.1 Study Design

It is a pilot study of a randomized, crossover clinical trial in which 28 participants, 14 healthy individuals, and 14 individuals with hypertension underwent taVNS at the tragus and cymba conchae in randomized sessions separated by a 1‐week interval.

### 2.2 Ethics

Participants were fully informed about the study procedures before providing written informed consent. This study was approved by the Research Ethics Committee of Nove de Julho University following Resolution 466/12 of the National Health Council, which governs research involving human subjects (CAAE 80780924.8.0000.5511). Additionally, the trial was registered and approved by the Brazilian Registry of Clinical Trials (https://ensaiosclinicos.gov.br/rg/RBR‐6tfbjjg) under the identifier REQ‐6tfbjjg.

### 2.3 Sample

Participants were recruited from the waiting list of the physiotherapy clinics at Nove de Julho University and through advertisements posted on social media platforms, including Facebook, Instagram, and WhatsApp.

Participants were included if they were adults over 18 years of age, of both genders, with a Mini‐Mental State Examination score above the expected cutoff for their education level (≥ 13 points for illiterate, ≥ 18 for low/medium education, and ≥ 26 for high education) (Bertolucci et al. 1994), with no contraindications for taVNS (e.g., absence of cochlear implants or metal implants at the stimulation site), no history of ischemic stroke, coronary artery disease, chronic obstructive or restrictive pulmonary disease, or peripheral arterial disease, no use of antihypertensive medications known to affect heart rate variability (e.g., beta‐blockers), not pregnant, and without a cardiac pacemaker. For the hypertension group, additional inclusion criteria were a medical diagnosis of stage I or II hypertension according to the Guidelines of the Brazilian Society of Hypertension (Barroso et al. 2021), use of up to three antihypertensive medications, and no history of cardiovascular diseases (except controlled hypertension), neuromuscular, endocrine, or metabolic diseases. For the healthy group, additional inclusion criteria were the absence of a history of cardiovascular diseases.

### 2.4 Interventions

#### 2.4.1 Transcutaneous Electrical Stimulation of the Vagus Nerve

**FIGURE 1:**
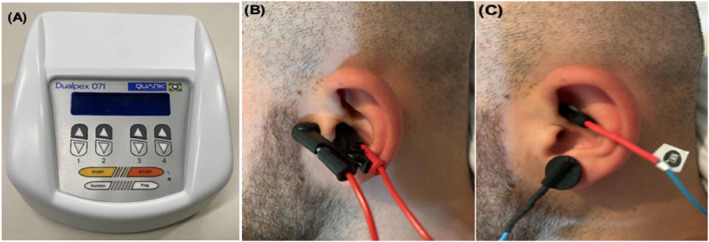
(A) Dualpex 071 device (Quark Medical Products). (B) Electrode placement for tragus stimulation using an ear clip containing both anode and cathode. (C) Electrode placement for cymba conchae stimulation, with the anode positioned on the cymba conchae and the cathode on the earlobe. *Source:* Authors’ own.

taVNS was applied with the multifunctional transcutaneous neuromuscular electrical stimulator, model DUALPEX 071, QUARK Medicine products (Anvisa registration no. 80079190022 (Figure 1A). Positioning of the electrodes for stimulation in the tragus is shown in Figure 1B, and positioning of the electrodes for stimulation in the cymba conche is presented in Figure 1C. The stimulation was performed unilaterally on the left side for three conditions.

To receive the stimulation, the participant was laid on a stretcher. During tragus stimulation, the electrode was secured using a clip containing both the anode and cathode (Figure 1B); for cymba conchae’s stimulation, two 3 mm silicone electrodes were used, one attached to the cymba conchae (anode) and the other to the earlobe (cathode) (Figure 1C), with the aid of a conductive gel to facilitate the propagation of the current, together with a micropore for better fixation of the electrode.

The current used was sinusoidal with a frequency of 30 Hz and a pulse width of 500 ms for a period of 30 min (de Moraes et al. 2023). The intensity ranged from 0.5 to 12 mA and was adjusted according to the participant's tolerance to avoid pain or muscle responses, along with maintaining stimulation within the sensory threshold (Trevizol et al. 2016). Participants were expected to feel only a tingling sensation. If the sensation faded during the 30‐min session, the researcher gradually increased the current until it was perceived again.

## 3 Outcomes

### 3.1 Primary Outcome

#### 3.1.1 Blood Pressure (BP)

Brachial BP was measured using an automatic device (Geratherm desktop AR, Geratherm Medical AG, Germany). Blood pressure measurements were obtained in the supine position, using the appropriate cuff size for the participant's arm circumference. Three consecutive measurements were performed, with one‐minute intervals between them. The final value was determined by the mean value of the last two measurements, as recommended by the Brazilian Society of Cardiology (Barroso et al. 2021). BP was measured at baseline, during stimulation and 10 min after the stimulation.

Blood pressure was assessed at three distinct time points: (i) baseline (after 10 min of rest in the supine position before stimulation), (ii) during stimulation (in the final minutes of the taVNS session—30 min), and (iii) post‐stimulation (10 min after the end of the intervention).

### 3.2 Secondary Outcome

#### 3.2.1 Cardiac Autonomic Modulation

Cardiac autonomic modulation was assessed through heart rate variability (HRV), calculated from RR intervals in accordance with the guidelines of the Task Force of the European Society of Cardiology and the North American Society of Pacing and Electrophysiology (1996). RR intervals were obtained using a heart rate monitor (V800, Polar Electro, Finland).

Data were exported to Kubios HRV software (version 2.0, Biosignal Analysis and Medical Imaging Group, Finland) for preprocessing and analysis. Recordings were visually inspected to identify artifacts, ectopic beats, and signal loss. Automatic artifact correction was then applied using the Kubios built‐in correction algorithm with a low‐to‐medium filter setting, as recommended for short‐term HRV analysis. Segments containing excessive noise, signal instability, or more than 5% corrected beats were excluded.

RR intervals were recorded continuously throughout the experimental protocol, which consisted of three phases: (i) baseline (10 min of rest prior to stimulation), (ii) stimulation (30 min of taVNS application), and (iii) recovery (15 min post‐stimulation). Participants remained in the supine position during data collection.

For HRV analysis, standardized 5‐min segments of stable RR interval data were extracted from each phase. Whenever possible, the final 5‐min segment of each phase was selected, provided that signal stability was ensured.

Frequency‐domain analysis included low‐frequency (LF) and high‐frequency (HF) components expressed in normalized units (n.u.), as well as the LF/HF ratio. Time‐domain indices included SDNN, SDANN, SDNNi, rMSSD (ms), and pNN50 (%). Nonlinear analysis included SD1, SD2, and sample entropy (SampEn). Outcomes were compared across baseline, stimulation, and recovery phases.

**FIGURE 2:**
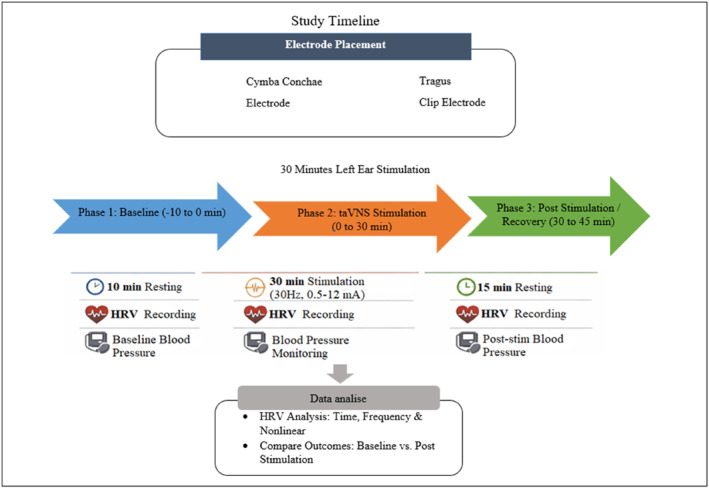
Study timeline.

A summary of the timeline can be seen in Figure 2.

### 3.3 Randomization and Blinding

Randomization of the intervention order (left tragus or left cymba concha) was performed using the Research Randomizer platform (www.randomizer.org). Number sets were generated to guarantee that each participant received a different stimulation site in the subsequent session. Data analysis was conducted by an evaluator blinded to group allocation, ensuring an impartial interpretation of results. Participants were not informed about the specific differences between the types or sites of stimulation (tragus vs. auricular concha). Thus, although participants perceived the sensory stimulation, they were unaware of the condition to which they were exposed during each session, which contributed to minimizing biases related to expectations or subjective perceptions.

### 3.4 Statistical Analysis of Data

Results are presented as mean and standard deviation for parametric data and as median and interquartile range for non‐parametric data. Delta (Δ) values were calculated to quantify changes from baseline to during and post‐intervention. Data were analyzed using generalized estimating equations, performed with the Statistical Package for the Social Sciences (SPSS), version 22.0 for Windows. A *p*‐value < 0.05 was considered significant.

Generalized estimating equations (GEE) were used to analyze repeated measures. Models were fitted in SPSS assuming a normal distribution with an identity link function. An unstructured working correlation matrix with robust standard errors was applied. The three‐way analyses included the factors time (baseline, stimulation, and post‐stimulation), stimulation site (cymba conche and tragus), and group (hypertensive and healthy). No additional covariates were included in the models.

## 4 Results

The study included 14 healthy and 14 hypertensive individuals, as detailed in the study flowcharts (Figures S1 and S2). Table 1 summarizes the participants' demographic characteristics.

**TABLE 1:** Demographic characteristics of healthy and hypertensive participants.

Characteristics	Healthy *N* = 14	Hypertensive *N* = 14	*P*
Age (years), mean (SD)	31 (7.3)	45 (12.1)	0.001
Gender (F/M)	5/9	9/5	0.141
Weight (kg), mean (SD)	77 (14.2)	69 (12.0)	0.116
Height (m), mean (SD)	1.72 (0.1)	2 (0.1)	0.543
BMI (kg/m^2^), mean (SD)	26 (3.8)	24 (3.7)	0.156
HR (bpm), mean (SD)	77 (16.4)	74 (4.6)	0.560
SBP (mmHg), mean (SD)	126 (8.6)	146 (6.9)	0.001
DBP (mmHg), mean (SD)	77 (12.1)	90 (6.4)	0.002
LF (nu) basal, mean (SD)	64.56 (18.36)	75.02 (15.72)	0.118
HF (nu) basal, mean (SD)	35.36 (18.34)	24.96 (15.71)	0.119
LF/HF basal, mean (SD)	3.13 (3.18)	4.42 (2.98)	0.277
Smoking history, *n* (%)	5 (35.7)	2 (14.28)	
Alcoholism, *n* (%)	8 (57.1)	5 (35.7)	
Time since diagnosis (years), mean (SD)		7 (6.1)	
Medications, *n* (%)			
Losartana		3 (21.4)	
Captropril		2 (14.3)	
Enalapril		2 (14.3)	
Espinolactona		1 (7.14)	
Telmisartana		1 (7.14)	
Hidralazina		2 (14.3)	
Furosemida		1 (7.14)	
Hidrocloritiazida		6 (42.9)	
Valsartana		1 (7.14)	
Olmesartana		1 (7.14)	

*Note:* Values presented in absolute frequency, mean and standard deviation (SD).

Abbreviations: BMI, body mass index; bpm, beats per minute; DBP, diastolic blood pressure; F, female; HF, high frequency; HR, heart rate; kg, kilogram; LF, low frequency; LF/HF, low frequency to high frequency ratio; M, male; m, meters; mmHg, millimeters per mercury; SBP, systolic blood pressure; SpO2, oxygen saturation.

The group of hypertensive individuals had a higher mean age and higher systolic and diastolic blood pressure levels (SBP and DBP) compared to the healthy group (*p* < 0.05); additionally, the proportion of female participants was higher in the hypertensive group than in the healthy group, although this difference was not statistically significant.

### 4.1 Primary Outcome—Blood Pressure

**FIGURE 3:**
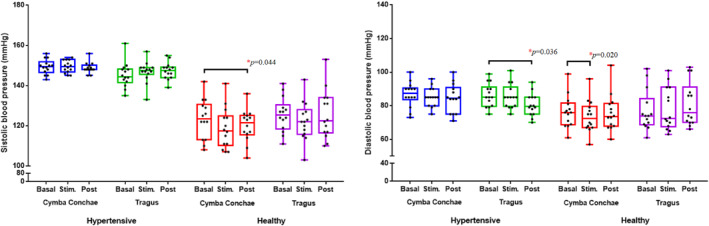
Effects of transcutaneous auricular vagus nerve stimulation (taVNS) at the cymba conchae and tragus on systolic blood pressure (SBP) and diastolic blood pressure (DBP) in hypertensive and healthy individuals, assessed at baseline, during, and post‐stimulation. Values are expressed in millimeters of mercury (mmHg). Boxplots show the median and interquartile range with individual data points overlaid. Analysis was performed using generalized estimating equations (GEE). *p* < 0.05.

Figure 3 illustrates the interaction effects between the groups (healthy and hypertensive) and assessment periods (pre, during stimulation and post‐stimulation). The complete results of the interaction between the SBP and DBP variables of hypertensive and healthy individuals are described in Table S1.

There was a significant interaction for PAS (*χ* ^2^ = 19.0 *p* = 0.001) and for PAD (*χ* ^2^ = 10.0; *p* = 0.038).

Stimulation of the cymba conchae did not alter BP in the hypertensive group (*p* > 0.05). In the healthy group, there was a significant decrease in systolic BP from baseline to post‐stimulation (*p* = 0.044) and in diastolic BP during stimulation (*p* = 0.020). Tragus stimulation reduced post‐stimulation diastolic BP (*p* = 0.036) and did not alter systolic BP in the hypertensive group (*p* < 0.05), whereas no effects were observed in the healthy group (*p* > 0.05).

### 4.2 Secondary Outcomes—Cardiac Autonomic Modulation

Table 2 presents the cardiac autonomic modulation before and after stimulation of the auricle and tragus in the healthy and hypertensive groups, respectively.

**TABLE 2:** Results of the interaction between variables of cardiac autonomic modulation in healthy and hypertensive individuals.

	Cymba conche (*n* = 14)			Tragus (*n* = 14)					
	Baseline	Post‐treatment	Δ Change	Baseline	Post‐treatment	Δ Change	Wald's chi‐square test	Degree of freedom	Interaction effect
SDNN									
Healthy	46 ± 19	60 ± 32^a^	7 ± 10	39 ± 12	49 ± 16^a^	5 ± 7	4.9	4	0.296
Hypertensive	29 ± 9	33 ± 12^a^	2 ± 3	30 ± 9	35 ± 14	3 ± 6			
RMMSD									
Healthy	44 ± 27	56 ± 43^a^	6 ± 12	36 ± 16	40 ± 17	2 ± 6	2.7	4	0.611
Hypertensive	21 ± 12	24 ± 15	2 ± 4	22 ± 9	26 ± 14	2 ± 5			
PNN50									
Healthy	21 ± 20	27 ± 23^a^	3 ± 5	16 ± 14	18 ± 14	1 ± 5	4.3	4	0.370
Hypertensive	5 ± 10	7 ± 13	1 ± 4	5 ± 6	8 ± 10	2 ± 4			
LFnu									
Healthy	65 ± 21	71 ± 19	3 ± 7	65 ± 18	74 ± 11^a^	5 ± 8	10.6	4	0.031^*^
Hypertensive	77 ± 11	73 ± 13	−2 ± 4	72 ± 16	71 ± 14	−1 ± 7			
HFnu									
Healthy	35 ± 21	28 ± 19	−3 ± 7	35 ± 18	26 ± 11^a^	−5 ± 8	10.6	4	0.031^*^
Hypertensive	23 ± 11	27 ± 13	2 ± 4	27 ± 16	29 ± 14	1 ± 7			
LF/HF									
Healthy	2.8 ± 1.8	4.0 ± 2.6^a^	0.6 ± 1	3.1 ± 3.2	3.6 ± 1.9	0.2 ± 1	12.9	4	0.012^*^
Hypertensive	4.5 ± 3.0	3.2 ± 1.3^a^	−0.7 ± 1	3.6 ± 2.1	3.1 ± 2.0	−0.2 ± 1			
SD1									
Healthy	31 ± 19	40 ± 31^a^	4 ± 8	26 ± 12	28 ± 12	1 ± 4	2.7	4	0.610
Hypertensive	15 ± 8	17 ± 11	1 ± 3	16 ± 6	19 ± 10	1 ± 4			
SD2									
Healthy	55 ± 22	74 ± 35^a^	9 ± 12	48 ± 14	64 ± 20^a^	3 ± 9	5.6	4	0.229
Hypertensive	38 ± 11	44 ± 15^a^	3 ± 3	39 ± 12	46 ± 17	3 ± 8			
SAMPEN									
Healthy	1.6 ± 0.4	1.6 ± 0.3	0.01 ± 0.1	1.6 ± 0.3	1.5 ± 0.3^a^	−0.07 ± 0.1	12.3	4	0.015^*^
Hypertensive	1.5 ± 0.1	1.5 ± 0.3	−0.03 ± 0.1	1.7 ± 0.2	1.4 ± 0.4^a^	−0.1 ± 0.1			

*Note:* Values expressed as mean and standard deviation. The test used was the Generalized Estimating Equations (GEE) test.

Abbreviations: PNN50, Percentage of adjacent RR intervals with a duration difference greater than 50 m; RMSSD, root mean square of successive differences; SAMPEN, Sample Entropy; SD1, standard deviation 1; SD2, standard deviation 2, SDDN, standard deviation of RR intervals.

^a^ Significant intragroup difference (baseline X post‐treatment) (*p* < 0.05).

^*^ *p* ≤ 0.05.

The generalized estimating equations (GEE) analysis revealed a significant group × time × stimulation site interaction effect. Significant interaction effects were observed for normalized spectral components and the non‐linear entropy measure. Specifically, significant interactions were found for LFnu (Wald *χ* ^2^ = 10.6, *p* = 0.031), HFnu (Wald *χ* ^2^ = 10.6, *p* = 0.031), and the LF/HF ratio (Wald *χ* ^2^ = 12.9, *p* = 0.012). In addition, SampEn also demonstrated a significant interaction effect (Wald *χ* ^2^ = 12.3, *p* = 0.015).

In hypertensive individuals, cymba conchae stimulation was associated with significant intragroup changes from baseline to post‐stimulation, including increases in SDNN (*p* = 0.001) and SD2 (*p* = 0.001), along with a reduction in the LF/HF ratio (*p* = 0.030). Tragus stimulation resulted in a significant reduction in SampEn (*p* = 0.004), without significant changes in other HRV parameters.

In healthy individuals, cymba conchae stimulation significantly increased SDNN (*p* = 0.004), RMSSD (*p* = 0.048), pNN50 (*p* = 0.031), LF/HF ratio (*p* = 0.014), SD1 (*p* = 0.048), and SD2 (*p* = 0.002). Tragus stimulation significantly increased SDNN (*p* = 0.001), LFnu (*p* = 0.022), and SD2 (*p* = 0.001), while significantly reducing HFnu (*p* = 0.022) and SampEn (*p* = 0.001).

## 5 Discussion

The objective of this study was to compare the acute effects of taVNS applied to the tragus and cymba conchae on blood pressure and heart rate variability in healthy and hypertensive subjects. The results showed that (i) cymba conchae stimulation reduced systolic BP post‐stimulation and diastolic BP during stimulation in healthy subjects; (ii) tragus stimulation reduced diastolic BP post‐stimulation; and (iii) neither intervention produced significant changes in cardiac autonomic modulation as assessed by HRV indices.

Consistent with previous evidence, hypertensive individuals exhibited higher systolic and diastolic blood pressure levels, as well as impaired baseline cardiac autonomic modulation characterized by reduced HRV indices. The absence of significant post‐intervention systolic blood pressure changes in this group may be related to impaired cardiovascular regulatory mechanisms commonly observed in hypertension, including reduced baroreflex sensitivity, increased sympathetic activity, and diminished vagal responsiveness.

Importantly, the hypertensive group was significantly older than the healthy group. Aging is a well‐established determinant of blood pressure regulation and autonomic function, being associated with increased arterial stiffness, progressive elevation of blood pressure, reduced baroreflex sensitivity, and decreased HRV (Fluckiger et al. 1999; Fanelli and Persu 2022). These effects tend to be more pronounced in individuals with hypertension (de Andrade et al. 2017), suggesting that the combined influence of aging and hypertension may further exacerbate autonomic dysfunction and potentially attenuate responsiveness to acute neuromodulation interventions.

Although baseline differences in LF and HF did not reach statistical significance, the tendency toward higher LF and lower HF values in the hypertensive group may be partially influenced by the older age of this group, as previously reported (Shah et al. 2023; Mondal and Bhattacharyya 2025). Furthermore, de Andrade et al. (2017) demonstrated a significant reduction in HRV among elderly hypertensive individuals, reinforcing the potential interaction between aging and hypertension on autonomic control. Therefore, the age difference between groups should be considered a potential confounding factor when interpreting the autonomic and hemodynamic findings of the present study.”

The effects observed exclusively during cymba conchae stimulation may be related to the denser vagal innervation of this region compared to the tragus, which presents mixed innervation and a lower density of vagus nerve fibers (Clancy et al. 2014). This anatomical characteristic suggests that cymba stimulation may more effectively recruit vagal afferent pathways, potentially modulating central circuits related to cognitive and affective functions. However, the absence of significant changes in HRV indices indicates that such effects were not detectable through conventional autonomic markers in the present acute protocol. It is possible that the hemodynamic responses observed reflect subtle or transient autonomic adjustments or involve central regulatory mechanisms not fully captured by standard HRV analysis. These findings reinforce the complexity of autonomic modulation in response to taVNS and highlight the potential need for more sensitive autonomic assessment methods.

The lack of significant changes observed in the present study may be related to the intensity and duration of the stimulation protocol. This interpretation is corroborated by Staley et al. (2020), who reported that five consecutive days of taVNS sessions at 25 Hz for 30 min, applied to the cymba conchae did not result in a significant reduction in blood pressure in individuals with hypertension. Together, these findings suggest that short‐term protocols may be insufficient to induce clinically meaningful cardiovascular adaptations and that stimulation parameters may require further optimization. The attenuated responsiveness observed in the hypertensive group may also be influenced by factors such as intervention duration, sample characteristics, or the chronicity of hypertension, reinforcing the need for prolonged and potentially more individualized stimulation protocols in this population.

In addition, future studies should consider incorporating more sensitive measures of cardiac autonomic modulation. Recently, Molefi et al. (2025) proposed novel metrics to assess the autonomic effects of taVNS using Symmetric Projection Attractor Reconstruction (SPAR) imaging derived from electrocardiographic (ECG) signals. These approaches appear capable of detecting subtle physiological changes in response to sham and taVNS that may not be fully captured by traditional HRV indices. The adoption of such advanced analytical methods may help refine the characterization of taVNS effects and clarify whether differential or potentially superior autonomic responses occur between stimulation sites, such as the cymba conchae and the tragus.

In the hypertensive group, systolic blood pressure remained unchanged during and after stimulation, suggesting limited acute responsiveness to vagal modulation under the present protocol. A significant reduction in diastolic blood pressure was observed from baseline to post‐stimulation with tragus stimulation, which may reflect delayed hemodynamic adjustments potentially mediated by autonomic mechanisms. These findings are consistent with the hypothesis that hypertension is associated with impaired baroreflex sensitivity and autonomic dysfunction, which may attenuate immediate cardiovascular responses while still allowing more gradual modulatory effects Parati et al. (2000). Furthermore, Antonino et al. (2017) demonstrated that acute improvements in cardiac baroreflex sensitivity induced by taVNS depend on the integrity of the autonomic nervous system, which is frequently compromised in hypertensive individuals. Therefore, although the immediate effects observed were modest, the present findings suggest that taVNS may have potential as an adjunctive strategy for autonomic modulation, particularly when applied using more prolonged or optimized stimulation protocols.

Healthy subjects did not exhibit significant changes in SBP or DBP during or after tragus stimulation. This finding may be related to a more balanced baseline autonomic profile in this group, which could attenuate the magnitude of observable hemodynamic responses to acute neuromodulation. Relatively preserved vagal tone and cardiovascular regulation may contribute to a reduced responsiveness to taVNS in healthy individuals, suggesting that the effects of stimulation are likely influenced by the pre‐existing functional state of the autonomic and cardiovasculares systems. This state‐dependent responsiveness may partly explain the more pronounced or differential effects observed in clinical populations compared with healthy subjects.

### 5.1 Effects of Transcutaneous Auricular Vagus Nerve Stimulation (taVNS) on Heart Rate Variability (HRV)

Overall, no significant changes were observed in global time‐domain HRV indices (SDNN, RMSSD, pNN50, SD1, and SD2), indicating that a single session of taVNS, under the applied conditions, was not sufficient to induce measurable alterations in overall RR interval variability. However, significant interactions were identified in normalized spectral components (LFnu, HFnu, and LF/HF ratios), as well as in sample entropy (SampEn), suggesting group‐dependent autonomic modulation.

In hypertensive individuals, a reduction in the LF/HF ratio accompanied by a modest increase in HFnu was observed following stimulation, suggesting a shift toward relative parasympathetic predominance or reduced sympathetic influence. Although these changes were not accompanied by significant increases in vagally mediated time‐domain indices (e.g., RMSSD or pNN50), the spectral profile suggests an acute modulatory effect on sympathovagal dynamics. Importantly, interpretation of the LF/HF ratio should be made cautiously given the ongoing debate regarding its validity as a direct marker of sympathovagal balance.

Conversely, healthy participants demonstrated an increase in LFnu and LF/HF ratio with a concomitant reduction in HFnu, reflecting a distinct autonomic response pattern. This response may represent a transient physiological adjustment in individuals with preserved baseline autonomic balance, reinforcing the notion that baseline autonomic state influences responsiveness to taVNS.

The absence of changes in global variability indices suggests that a single session may not be sufficient to induce robust alterations in HRV amplitude, corroborating previous findings indicating that repeated or prolonged stimulation protocols may be required for clinically meaningful effects (Staley et al. 2020; de Moraes et al. 2023).

Regarding stimulation sites, both cymba conchae and tragus elicited similar response patterns, with no clear evidence of regional superiority. Although anatomical differences in vagal innervation density have been described (Peuker and Filler 2002; Frangos et al. 2015; Corrêa et al. 2022), the observed effects were primarily group‐dependent rather than site‐specific.

The reduction in SampEn, particularly in hypertensive participants, may indicate short‐term reorganization of autonomic control complexity following stimulation. Given the acute nature of the intervention, these findings should be interpreted cautiously. Additionally, no significant order effects were identified, indicating that the stimulation sequence did not influence the outcomes.

Collectively, these findings suggest that acute taVNS does not modify global HRV magnitude but may transiently modulate spectral components associated with sympathovagal dynamics, with differential effects depending on baseline autonomic status.

### 5.2 Study Limitations

This study has several limitations that should be acknowledged. The smaller sample size, single‐center recruitment, and short follow‐up period may limit the generalizability of the findings and preclude conclusions regarding the long‐term effects of auricular stimulation.

In addition, the hypertensive group was significantly older than the healthy group. Given that age is a recognized determinant of blood pressure regulation and cardiac autonomic modulation, this imbalance may have acted as a confounding factor in the interpretation of hemodynamic and heart rate variability outcomes. Thus, part of the observed autonomic responses may reflect age‐related cardiovascular changes rather than hypertension per se. Future studies with age‐matched groups are warranted.

Stimulation intensity was individually adjusted to sensory threshold but not systematically recorded by group or stimulation site, precluding subgroup‐specific analyses, although a standardized protocol was applied to all participants.

Finally, the absence of a sham control group limits causal inference regarding vagal nerve activation. Despite the use of internal comparisons and delta analyses to reduce interindividual variability, randomized sham‐controlled studies with larger samples are needed to confirm these findings.

### 5.3 Implications for Physical Therapy Practice

taVNS may represent a promising strategy in physical therapy for hypertension by modulating the autonomic nervous system, enhancing parasympathetic activity and suppressing sympathetic activity, thereby contributing to reductions in blood pressure and heart rate. Furthermore, it promotes stress and anxiety reduction, improves cardiovascular function, and promotes vascular health (Farmer et al. 2020; Carandina et al. 2021; Yap et al. 2020; Bremner et al. 2020). Because it is safe, non‐invasive, and easy to apply, taVNS can complement conventional physical therapy approaches, enhancing hypertension control. These findings reinforce the need for future studies to clarify its mechanisms and establish its clinical relevance.

## 6 Conclusion

This study is the first to compare the acute effects of taVNS applied to the cymba conchae and tragus on heart rate variability and blood pressure in hypertensive and healthy individuals. A single stimulation session did not produce significant changes in global HRV indices or blood pressure, regardless of the stimulation site. However, group‐dependent modulation was observed in specific spectral HRV components, suggesting transient autonomic adjustments rather than robust cardiovascular effects.

These findings highlight that acute taVNS may not be sufficient to induce clinically meaningful autonomic or hemodynamic alterations. Prolonged, repeated, or individualized stimulation protocols may be required to achieve sustained therapeutic outcomes. Further research with larger sample sizes and rigorously controlled designs is necessary to confirm these preliminary findings and clarify the clinical relevance of taVNS in hypertensive populations.

The absence of order effects further strengthens the robustness of the observed patterns.

## Funding

This work was supported by the Coordination for the Improvement of Higher Education Personnel (CAPES) with funding from the first and third authors [process numbers 88887.820515/2023‐00 and 88887.835995/2023‐00], respectively.

## Ethics Statement

This study was approved by the Research Ethics Committee of Nove de Julho University following Resolution 466/12 of the National Health Council, which governs research involving human subjects (CAAE: 80780924.8.0000.5511), approval date (October 2, 2024). Additionally, the trial was registered and approved by the Brazilian Registry of Clinical Trials (https://ensaiosclinicos.gov.br/rg/RBR‐6tfbjjg) under the identifier REQ‐6tfbjjg.

## Consent

Informed consent was obtained from all the individual participants included in the study.

## Conflicts of Interest

The authors declare no conflicts of interest.

## Supporting information


**Figure S1:** Flowchart of the study of healthy individuals.

**Figure S2:** Flowchart of the study of hypertensive participants.

**Table S1:** Interaction effects of transcutaneous auricular vagus nerve stimulation on systolic and diastolic blood pressure in healthy and hypertensive participants.

## Data Availability

The data were analyzed during the current study, and the dataset used is available from the corresponding author upon reasonable request.
