# Supplementary material for: Acute Effect of Transcutaneous Auricular Vagus Nerve Stimulation in Two Different Locations on Blood Pressure and Cardiac Autonomic Modulation in Healthy and Hypertensive Individuals: Pilot Study of a Randomized Crossover Clinical Trial
Source: Physiother Res Int. 2026 Apr 7;31(2):e70209. doi: 10.1002/pri.70209 (PMC13054635; doi:10.1002/pri.70209)
Supplement: Supplementary file 2 — Figure S2: Flowchart of the study of hypertensive participants. [file PRI-31-e70209-s002.docx]

taVNS tragus

HRV Assessment (N=8)

taVNS cymba conchae

HRV Assessment (N=6)

taVNS tragus

HRV Assessment (N=6)

taVNS cymba conchae

HRV Assessment (N=8)

**Figure S2:** Flowchart of the study of hypertensive participants. Transcutaneous auricular vagus nerve stimulation (taVNS), Blood pressure (BP), heart rate (HR), oxygen saturation (SpO2), heart rate variability (HRV).

Excluded (N=0)

Do not meet the inclusion criteria (N=0)

Withdrew from participation (N=0)

Other reasons (N=0)

Analized (N= 14)

incinçcInc

Interval 1 week

incinçcInc

Post-Evaluation

BP, HR. SpO2, HRV (N=14)

crossover

Pre-assessment

BP, HR, SpO2, HRV (N=14)

Post-Evaluation

BP, HR. SpO2, HRV (N= 14)

Randomization

Pre-assessment

BP, HR, SpO2, HRV (N=14)

Evaluated for eligibility (N=14)
