# Supplementary material for: Acute Effect of Transcutaneous Auricular Vagus Nerve Stimulation in Two Different Locations on Blood Pressure and Cardiac Autonomic Modulation in Healthy and Hypertensive Individuals: Pilot Study of a Randomized Crossover Clinical Trial
Source: Physiother Res Int. 2026 Apr 7;31(2):e70209. doi: 10.1002/pri.70209 (PMC13054635; doi:10.1002/pri.70209)
Supplement: Supplementary file 3 — Table S1: Interaction effects of transcutaneous auricular vagus nerve stimulation on systolic and diastolic blood pressure in healthy and hypertensive participants. [file PRI-31-e70209-s003.docx]

Table S1: Interaction Effects of Transcutaneous Auricular Vagus Nerve

Stimulation on Systolic and Diastolic Blood Pressure in Healthy and

Hypertensive Participants

|  |  | **Wald's chi-square test** | **Degree of freedom** | **Interaction effect** |
| --- | --- | --- | --- | --- |
| **SBP** |  |  |  |  |
| Healthy |  | 19.0 | 4 | 0.001* |
| Hypertensive |  |  |  |  |
| **DBP** |  |  |  |  |
| Healthy |  | 10.0 | 4 | 0.038* |
| Hypertensive |  |  |  |  |
|  |  |  |  |  |

Legend: SBP = systolic blood pressure; DBP = diastolic blood pressure.

Results derived from generalized estimating equation (GEE)models. *p ≤ 0.05.
